# Supplementary material for: Measuring the impact of climate change on potato production in Bangladesh using Bayesian Hierarchical Spatial-temporal modeling
Source: PLoS One. 2022 Nov 22;17(11):e0277933. doi: 10.1371/journal.pone.0277933 (PMC9681075; doi:10.1371/journal.pone.0277933)
Supplement: Attachment — Submitted filename: Response to Reviewers.pdf [file pone.0277933.s001.pdf]

Author responses to the review comments:

We would like to express our sincere gratitude to the two reviewers and the Academic Editor for their valuable comments. We have considered all the comments made by the reviewers and thoroughly revised and formatted the manuscript accordingly. A detailed response to each of the comments is provided in the table below:

**Academic Editor comments:**	**Response**	**Note**
Please include the following items when submitting your revised manuscript:<br>A rebuttal letter that responds to each point raised by the academic editor and reviewer(s). You should upload this letter as a separate file labeled 'Response to Reviewers'.<br>A marked-up copy of your manuscript that highlights changes made to the original version. You should upload this as a separate file labeled 'Revised Manuscript with Track Changes'.<br>An unmarked version of your revised paper without tracked changes. You should upload this as a separate file labeled 'Manuscript'.<br><br>If you would like to make changes to your financial disclosure, please include your updated statement in your cover letter. Guidelines for resubmitting your figure files are available below the reviewer comments at the end of this letter.	Thank you very much. The required files are submitted through the submission system.<br><br>We include all required information in the cover letter.	Revised texts are in red color.
**Journal Requirements:**	**Response**	**Note**
1. Please ensure that your manuscript meets PLOS ONE's style requirements, including those for file naming. The PLOS ONE style templates can be found at https://journals.plos.org/plosone/s/file?id=wjVg/PLOSOne_formatting_sample_main_body.pdf and https://journals.plos.org/plosone/s/file?id=ba62/PLOSOne_formatting_sample_title_authors_affiliations.pdf.	Many thanks. The manuscript is revised according to PLOS ONE’s style.<br><br>All necessary files are uploaded to the system of the journal.	Revised texts are in red color.<br>Page: 1-16
2. We suggest you thoroughly copyedit your manuscript for language usage, spelling, and grammar. If you do not know anyone who can help you do this, you may wish to consider employing a professional scientific editing service.<br><br>Whilst you may use any professional scientific editing service of your choice, PLOS has partnered with both American Journal Experts (AJE) and Editage to provide discounted services to PLOS authors. Both organizations have experience helping authors meet PLOS	Thanks for raising these points. We revised the whole manuscript as per your comments.<br><br>The whole manuscript is revised in light of grammatical mistakes and typos.<br><br>A clean version of the manuscript and track changed version is uploaded to the journal system.	Revised texts are in red color.<br>Page: 1-16

guidelines and can provide language editing, translation, manuscript formatting, and figure formatting to ensure your manuscript meets our submission guidelines. To take advantage of our partnership with AJE, visit the AJE website (http://learn.aje.com/plos/) for a 15% discount off AJE services. To take advantage of our partnership with Editage, visit the Editage website (www.editage.com) and enter referral code PLOSEDIT for a 15% discount off Editage services. If the PLOS editorial team finds any language issues in text that either AJE or Editage has edited, the service provider will re-edit the text for free.<br>Upon resubmission, please provide the following:<br>The name of the colleague or the details of the professional service that edited your manuscript<br>A copy of your manuscript showing your changes by either highlighting them or using track changes (uploaded as a *supporting information* file)<br>A clean copy of the edited manuscript (uploaded as the new *manuscript* file).		
3. We note that you have indicated that data from this study are available upon request. PLOS only allows data to be available upon request if there are legal or ethical restrictions on sharing data publicly. For more information on unacceptable data access restrictions, please see http://journals.plos.org/plosone/s/data-availability#loc-unacceptable-data-access-restrictions.<br>In your revised cover letter, please address the following prompts:<br>a) If there are ethical or legal restrictions on sharing a de-identified data set, please explain them in detail (e.g., data contain potentially sensitive information, data are owned by a third-party organization, etc.) and who has imposed them (e.g., an ethics committee). Please also provide contact information for a data access committee, ethics committee, or other institutional body to which data requests may be sent.<br>b) If there are no restrictions, please upload the minimal anonymized data set necessary to replicate your study findings as either Supporting Information files or to a stable, public repository and provide us with the relevant URLs, DOIs, or accession numbers. For a list of acceptable repositories, please see	Thanks. We have revised the data availability statement.<br><br>This study is based on the secondary data that is freely available in the websites of the Bangladesh Bureau of Statistics (http://bbs.portal.gov.bd/sites/default/files/files/bbs.portal.gov.bd/page/b343a8b4_956b_45ca_872f_4cf9b2f1a6e0/45%20years%20Major%20Crops.pdf) and Bangladesh Agricultural Research Council (https://bbs.portal.gov.bd/site/page/453af260-6aea-4331-b4a5-7b66fe63ba61/-).	Revised texts are in red color.<br>Page: 16

http://journals.plos.org/plosone/s/data-availability#loc-recommended-repositories.<br>We will update your Data Availability statement on your behalf to reflect the information you provide.		
4. We note that Figures 1, 3 and 4 in your submission contain [map/satellite] images which may be copyrighted. All PLOS content is published under the Creative Commons Attribution License (CC BY 4.0), which means that the manuscript, images, and Supporting Information files will be freely available online, and any third party is permitted to access, download, copy, distribute, and use these materials in any way, even commercially, with proper attribution. For these reasons, we cannot publish previously copyrighted maps or satellite images created using proprietary data, such as Google software (Google Maps, Street View, and Earth). For more information, see our copyright guidelines: http://journals.plos.org/plosone/s/licenses-and-copyright.<br>We require you to either (1) present written permission from the copyright holder to publish these figures specifically under the CC BY 4.0 license, or (2) remove the figures from your submission:<br>a) You may seek permission from the original copyright holder of Figures 1, 3 and 4 to publish the content specifically under the CC BY 4.0 license.<br>We recommend that you contact the original copyright holder with the Content Permission Form (http://journals.plos.org/plosone/s/file?id=7c09/content-permission-form.pdf) and the following text:<br>“I request permission for the open-access journal PLOS ONE to publish XXX under the Creative Commons Attribution License (CCAL) CC BY 4.0 (http://creativecommons.org/licenses/by/4.0/). Please be aware that this license allows unrestricted use and distribution, even commercially, by third parties. Please reply and provide explicit written permission to publish XXX under a CC BY license and complete the attached form.”<br>Please upload the completed Content Permission Form or other proof of granted permissions as an "Other" file with your submission.	Thank you very much for your concern on this point.<br><br>We ensure you that all maps are produced by using R-coding written by authors. All of them are the author’s own work. No maps were taken from any other sources.	

In the figure caption of the copyrighted figure, please include the following text: "Reprinted from [ref] under a CC BY license, with permission from [name of publisher], original copyright [original copyright year]."<br>b) If you are unable to obtain permission from the original copyright holder to publish these figures under the CC BY 4.0 license or if the copyright holder's requirements are incompatible with the CC BY 4.0 license, please either i) remove the figure or ii) supply a replacement figure that complies with the CC BY 4.0 license. Please check copyright information on all replacement figures and update the figure caption with source information. If applicable, please specify in the figure caption text when a figure is similar but not identical to the original image and is therefore for illustrative purposes only.<br>The following resources for replacing copyrighted map figures may be helpful:<br>USGS National Map Viewer (public domain): http://viewer.nationalmap.gov/viewer/<br>The Gateway to Astronaut Photography of Earth (public domain): http://eol.jsc.nasa.gov/sseop/clickmap/<br>Maps at the CIA (public domain): https://www.cia.gov/library/publications/the-world-factbook/index.html and https://www.cia.gov/library/publications/cia-maps-publications/index.html<br>NASA Earth Observatory (public domain): http://earthobservatory.nasa.gov/<br>Landsat: http://landsat.visibleearth.nasa.gov/<br>USGS EROS (Earth Resources Observatory and Science (EROS) Center) (public domain): http://eros.usgs.gov/#<br>Natural Earth (public domain): http://www.naturalearthdata.com/.		
**Additional Editor Comments:**	**Response**	**Note**
The manuscript entitled ""Measuring the Impact of Climate Change on Potato Production in Bangladesh using Bayesian Hierarchical Spatio-temporal Modelling" (PONE-D-22-21496)" is very important for potato production. Here the authors given a good impression about the current situation of Measuring the Impact of Climate Change on Potato Production in Bangladesh. The findings revealed that there is a potential impact of climate change on potato production in	Thank you very much. We have revised the Introduction section as per comments.	Revised texts are in red color.<br>Page: 2-4

Bangladesh. Bayesian Spatiotemporal modelling for Linear, ANOVA, and Auto-Regressive models was used to find the best fit compared with the independent Error Bayesian model for statistical analysis. However, the authors need to be focus on the following issues for modification-<br>1. Some specific previous studies on correlation between climate factors and its effect on potato crop (if available) may be included or mentioned in the introduction part.		
2. English should be checked by any native speaker.	Thanks. The whole manuscript is revised to fix grammatical issues and typos.	Revised texts are in red color.<br>Page: 1-16
3. Modification is required for proper synchronization of figures and legends in journal format.	Thank you very much. All figures and legends are modified as per requirement.<br>All high resolute figures are uploaded to the journal system.	Revised texts are in red color.<br>Page: 7-13
4. Resolution of the images use here should be increased.	Thank you very much for highlighted this point. All high resolute figures are uploaded to the journal system separately.	
5. Needs clearly defined objectives with proper deliverable outcome in details.<br>Concerning the above-mentioned issues, I am suggesting that this manuscript can be resubmitted as a major revision to this journal.	Thanks. We have revised the manuscript and submitted it to the journal system.	
**Reviewer 1 comments:**	**Response**	**Note**
1. In line no -53 , the font size has to be rectified.	Thank you very much for your comments and feedback. We revise it.	Revised texts are in red color.<br>Page: 3
2. Some specific previous studies on correlation between climate factors and its effect on potato crop (if available) may be included or mentioned in the introduction part.	Thanks. We appreciate this comment. We have revised the Introduction section as per your comments.	Revised texts are in red color.<br>Page: 2-4
**Reviewer 2 comments:**	**Response**	**Note**
The author has given a good impression about the current situation of Measuring the Impact of Climate Change on Potato Production in Bangladesh. The findings revealed that there is a potential impact of climate change on potato production in Bangladesh. Bayesian Spatiotemporal modelling for Linear, ANOVA, and Auto-Regressive models was used to find the best fit compared with the independent Error Bayesian model for statistical analysis.	Thank you very much for your valuable comment and suggestions that help us improve the manuscript's quality. We have revised the manuscript as per your comments.<br><br>The whole manuscript is revised to fix grammatical issues and typos.	Revised texts are in red color.<br>Page: 1-16

1. English modification has to be done in proper manner.		
2. Ligand should be added in figure 1. Addition of standard deviation should added in Fig 1.	Thanks. Actually, we consider the yearly potato production in Bangladesh. It is a single time series data, therefore, we think that to calculate the standard deviation is not possible here. In the left panel of Fig 1, we visualized the total potato production of Bangladesh by a time series plot and in the right panel, we want to show the spatial distribution of the potato production.	Revised texts are in red color. Page: 7
3. Explanation of * should be clearly written as figure ligand in Fig 2.	Thanks. We add the explanation of * in legend of Fig 2.	Revised texts are in red color. Page: 8
4. Clarity of the Fig 3 should be increased.	Thank you very much. We appreciate this comment.<br>All high resolute figures are uploaded to the journal system.	
5. Discussion part must be more elaborated.	Thanks. The Discussion section is revised as per your comment.	Revised texts are in red color. Page: 13-16

Finally, the revised manuscript has been produced following the valuable comments and suggestions of the reviewers. Once again, we would like to thank the reviewers for their sincere dedication, professional insights, and earnest cooperation in reviewing the manuscript.
