# Supplementary material for: Measuring the impact of climate change on potato production in Bangladesh using Bayesian Hierarchical Spatial-temporal modeling
Source: PLoS One. 2022 Nov 22;17(11):e0277933. doi: 10.1371/journal.pone.0277933 (PMC9681075; doi:10.1371/journal.pone.0277933)
Supplement: Attachment — Submitted filename: Response to Reviewers.docx [file pone.0277933.s002.docx]

**Author responses to the review comments:**

We would like to express our sincere gratitude to the two reviewers and the Academic Editor for their valuable comments. We have considered all the comments made by the reviewers and thoroughly revised and formatted the manuscript accordingly. A detailed response to each of the comments is provided in the table below:

| **Academic Editor comments:** | **Response** | **Note** |
| --- | --- | --- |
| Please include the following items when submitting your revised manuscript:  A rebuttal letter that responds to each point raised by the academic editor and reviewer(s). You should upload this letter as a separate file labeled 'Response to Reviewers'.  A marked-up copy of your manuscript that highlights changes made to the original version. You should upload this as a separate file labeled 'Revised Manuscript with Track Changes'.  An unmarked version of your revised paper without tracked changes. You should upload this as a separate file labeled 'Manuscript'.  If you would like to make changes to your financial disclosure, please include your updated statement in your cover letter. Guidelines for resubmitting your figure files are available below the reviewer comments at the end of this letter. | Thank you very much. The required files are submitted through the submission system.  We include all required information in the cover letter. | Revised texts are in red color. |
| **Journal Requirements:** | **Response** | **Note** |
| Please review your reference list to ensure that it is complete and correct. If you have cited papers that have been retracted, please include the rationale for doing so in the manuscript text, or remove these references and replace them with relevant current references. Any changes to the reference list should be mentioned in the rebuttal letter that accompanies your revised manuscript. If you need to cite a retracted article, indicate the article’s retracted status in the References list and also include a citation and full reference for the retraction notice. | Many thanks. We checked all the references and ensure that all of them are complete and correct. |  |
| **Additional Editor Comments:** | **Response** | **Note** |
| After reevaluating this revised manuscript the following revisions can be taken into consideration-  1. Modifying English Language specially in introduction and conclusion section. | Thank you very much. We have revised the Introduction and Conclusion sections as per comments.  Moreover, the whole manuscript is revised to fix grammatical issues and typos. | Revised texts are in red color.  Page: 1-13 |
| 2. Give some justification how this model can be implemented to another agro climatic environment. i.e. Mention global impact of using this model. | Thanks. We add this in the last part of the Introduction section. | Revised texts are in red color.  Page: 4 |
| **Reviewer’s comments:** | **Response** | **Note** |
| While revising your submission, please upload your figure files to the Preflight Analysis and Conversion Engine (PACE) digital diagnostic tool, https://pacev2.apexcovantage.com/. PACE helps ensure that figures meet PLOS requirements. To use PACE, you must first register as a user. Registration is free. Then, login and navigate to the UPLOAD tab, where you will find detailed instructions on how to use the tool. If you encounter any issues or have any questions when using PACE, please email PLOS at figures@plos.org. Please note that Supporting Information files do not need this step. | Thank you very much for your comments and feedback. We already checked all figures using PACE at the time of submission of the revised version of the manuscript (PONE-D-22-21496R1). |  |

Finally, the revised manuscript has been produced following the valuable comments and suggestions of the reviewers. Once again, we would like to thank the reviewers for their sincere dedication, professional insights, and earnest cooperation in reviewing the manuscript.
